# Photon-counting detector CT with an ultra-low-dose contrast media to diagnose a renal pseudoaneurysm: A case report

**DOI:** 10.1016/j.radcr.2024.05.077

**Published:** 2024-06-15

**Authors:** Takayuki Noro, Yoshinao Ojio, Misugi Urano, Kengo Ohta, Kazushi Suzuki, Takafumi Sato, Keita Nakayama, Shota Ohba, Tatsuya Kawai, Toshihide Itoh, Akio Hiwatashi

**Affiliations:** a Department of Radiology, Nagoya City University Graduate School of Medical Sciences, Mizuho-ku, Nagoya, Japan; b Department of Radiology, Kariya Toyota General Hospital, Kariya, Japan; c CT-Research and Collaboration, Siemens Healthineers, Tokyo, Japan

**Keywords:** Photon-counting CT, Computed tomography angiography, Contrast media, Aneurysm, False, Renal insufficiency, Chronic

## Abstract

A 75-year-old male, weighing 71 kg, was admitted to our institution with anemia related to a subcapsular hematoma after accidental extraction of a nephrostomy catheter. While the patient exhibited the progression of chronic kidney disease, he was not yet on dialysis. His serum creatinine level increased to 6.8 mg/dL, with an estimated glomerular filtration rate of 7.4 mL/min/1.73 m^2^. Radiologists planned contrast-enhanced photon-counting detector CT (PCD-CT) with an ultra-low-dose contrast media to mitigate the impact on renal function. The contrast media dosage was set at 7.4 gI, which was 82.6% lower that used in the standard protocol for a male weighing 71 kg. Non-contrast-enhanced PCD-CT identified a low-density nodular area within the renal subcapsular hematoma. Contrast-enhanced PCD-CT revealed contrast enhancement in both the early and late phases corresponding to the nodular area. On virtual monoenergetic images, the renal pseudoaneurysm was most clearly delineated at 40 keV. Following the diagnosis of a pseudoaneurysm, transcatheter arterial coil embolization was performed. No subsequent progression of anemia or the deterioration of renal function was observed, showcasing the potential of ultra-low-dose contrast-enhanced PCD-CT for the detection of small vascular abnormalities while minimizing adverse effects on renal function.

## Introduction

Contrast-enhanced CT is necessary for diagnosing a renal pseudoaneurysm and assessing indications for transcatheter arterial embolization (TAE) [1]. The administration of iodinated contrast media to patients with chronic kidney disease (CKD) is associated with the risk of contrast-induced acute kidney injury (CI-AKI) [2,3]. Although using the minimum dose of contrast media helps mitigate the risk of CI-AKI, it may result in poor image contrast, potentially impeding an accurate diagnosis on contrast-enhanced CT. Photon-counting detector CT (PCD-CT) generates multi-kilo electron volt (keV) virtual monochromatic X-ray images (VMI) to improve image contrast utilizing X-ray spectral data while maintaining a lower noise level than conventional energy-integrating detector CT (EID-CT) [4,5]. The present study describes a case of a 75-year-old man with a renal pseudoaneurysm within the subcapsular hematoma that was successfully visualized using an ultra-low-dose contrast media on PCD-CT.

## Case report

A 75-year-old male weighing 71 kg with CKD not yet on dialysis, who had a history of right renal nephrostomy due to repetitive hydronephrosis after an ileal conduit diversion, developed lightheadedness after the nephrostomy catheter was accidentally extracted. Blood test results showed anemia (hemoglobin level: 6.7 g/dL), and non-enhanced CT revealed a right renal subcapsular hematoma. Since an infusion of 2 units of red blood cells did not increase the level of hemoglobin, the patient was transferred and admitted to our hospital the following day. Blood tests in our hospital showed a hemoglobin level of 6.9 g/dL and creatinine level of 6.8 mg/dL, calculating an estimated glomerular filtration rate (eGFR) of 7.4 mL/min/1.73 m^2^.

Urologists suspected active bleeding and scheduled contrast-enhanced CT. Radiologists planned a contrast protocol prior to CT in consideration of the progression of CKD. We usually use contrast media of 0.6 gI/kg for the patients suspected to be in a state of active bleeding. Based on the calculation, patients weighing 71 kg are required 42.6 gI. However, we had to reduce the dose to prevent CI-CKD. To reduce the contrast media to less than 1.1 × eGFR, by referring to previous studies [2,3], the radiologist set the dosage to 7.4 gI, which was 82.6% lower than that used in the standard protocol in our hospital.

**Fig. 1:**
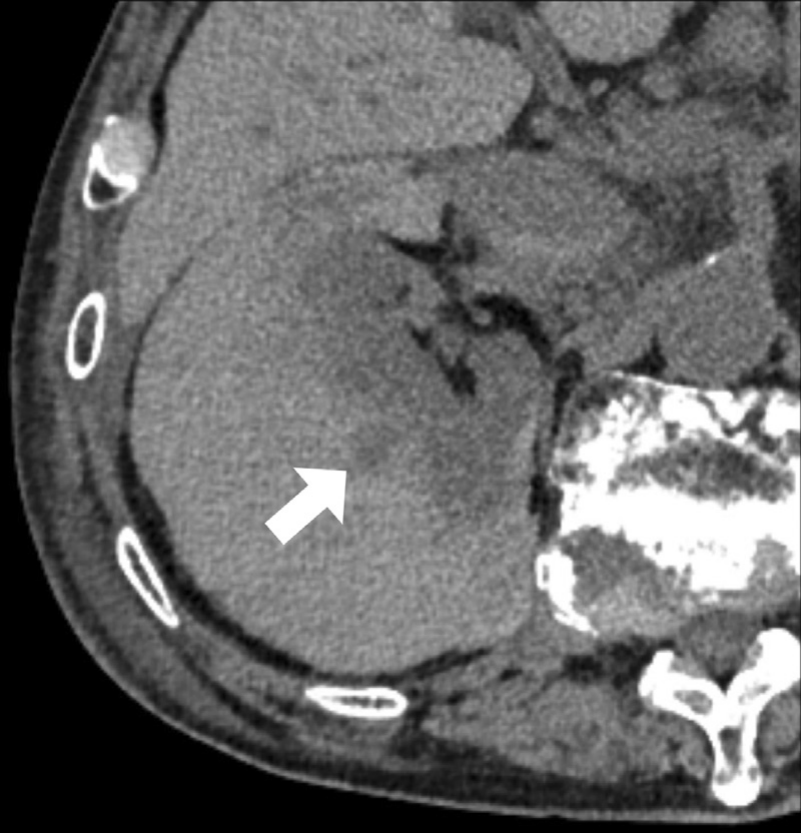
Non-enhanced PCD-CT shows a nodular low-density area (arrow) within the high-density area corresponding to the subcapsular hematoma surrounding the right kidney.

**Fig. 2:**
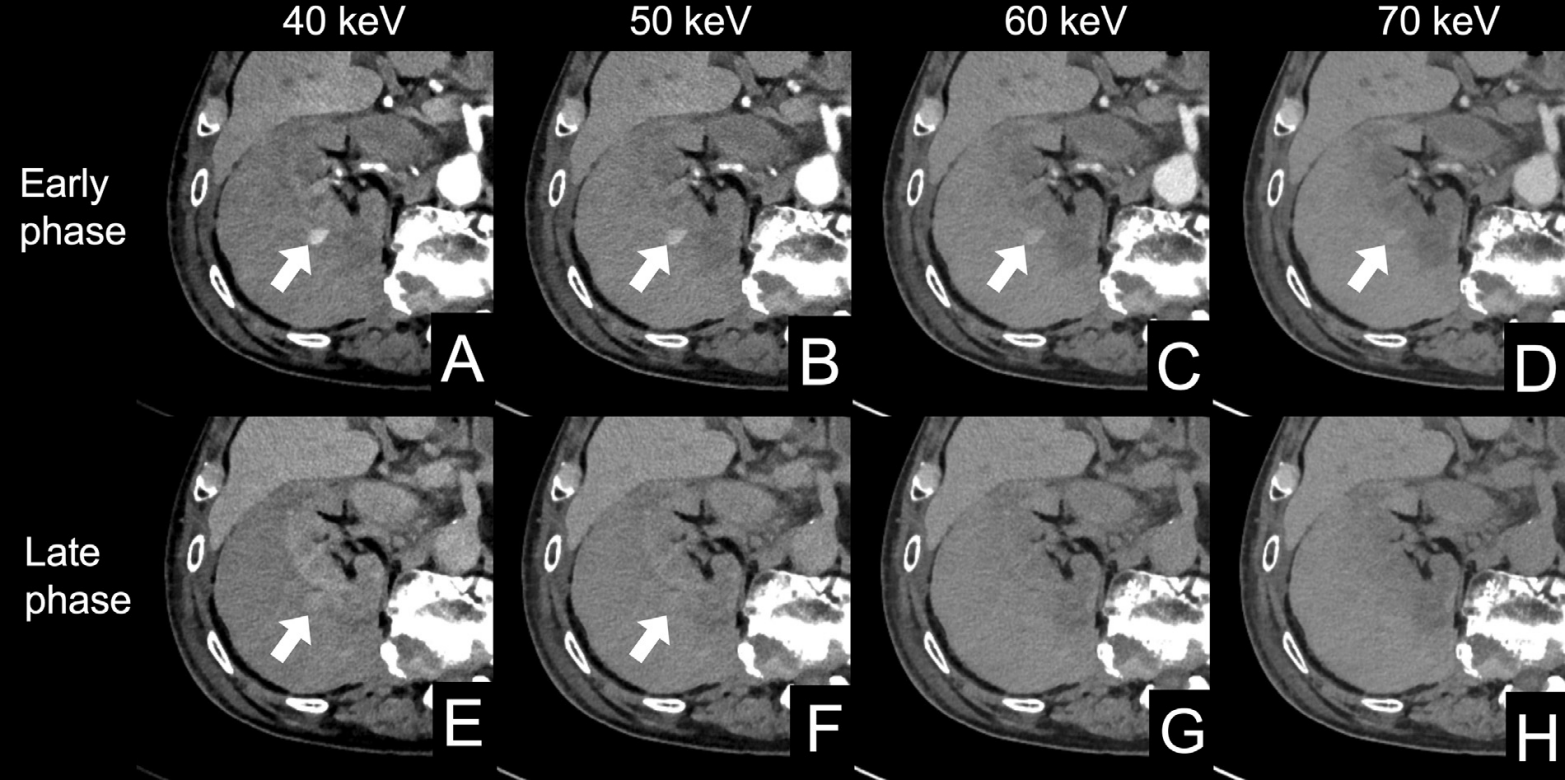
Comparison of VMI of the renal pseudoaneurysm at different keVs in early (A-D) and late (E-H) phases. The early phase at 40-70 keV (A-D) and late phase at 40, 50 keV (E, F) of contrast-enhanced images show an enhancing area corresponding to the nodular low-density area on non-enhanced CT (arrows). The enhancement of the renal pseudoaneurysm is delineated more at 40 keV than at higher keVs. On late phase contrast-enhanced images, the renal pseudoaneurysm is undetectable at 60 and 70 keV (G, H).

**Fig. 3:**
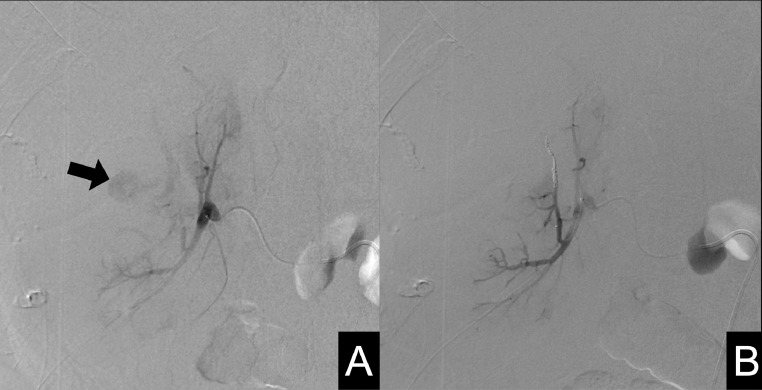
Angiograms of the right renal artery. (A) A right renal artery angiogram shows the pseudoaneurysm (arrow). (B) A postembolization arteriogram shows the disappearance of the pseudoaneurysm.

CT was performed using a PCD-CT system (NAEOTOM Alpha, Siemens Healthineers, Forchheim, Germany) in the spiral acquisition mode with 0.4 mm × 144, a spiral pitch of 0.8, tube voltage of 120 kV, and CT dose index volume of 57.2 (mGy). The scan range was from the diaphragm to the pelvis. After unenhanced CT, intravenous access was secured by placing a 22-gauge intravenous line in the antecubital vein. A bolus administration of the contrast media was performed by injecting 20 ml of 370 mgI/mL non-ionic iodine contrast media (Iopamiron 370, Bayer, Nordrhein-Westfalen, Germany) followed by 60 mL of saline at a rate of 4.0 mL/second using a power injector. The bolus-tracking technique was used to trigger the start of image acquisition, with region of interest (ROI) placement in the descending aorta. The ROI threshold was set at 100 Hounsfield units. The delay times of the scans for the arterial and delayed phases were 5 and 90 seconds, respectively. Spectral CT scans reconstructed with slice thicknesses of 1 and 5 mm, a Qr40 kernel, and iterative reconstruction strength of 3, were sent to a dedicated workstation (syngo. via VB60A, Siemens Healthineers, Forchheim, Germany) to generate virtual monoenergetic images (VMI) for the early and late phases with a slice thickness of 1 mm in 1-mm increments at 40–70 keV in 10 keV. Non-contrast-enhanced images revealed a low-density nodular area within the subcapsular hematoma (Fig. 1). Alternatively, contrast-enhanced images revealed an enhancement in the early and late phases corresponding to the nodular area, diagnostic of a pseudoaneurysm. On VMI, the pseudoaneurysm was most clearly visible at 40 keV (Fig. 2). TAE with 20 mL of 300 mgI/mL (6.0 gI) non-ionic iodine contrast media (Iopamidol 300, Fuji Pharma, Tokyo, Japan), doubling the volume of the contrast media through a 1:1 saline dilution, was planned. The pseudoaneurysm was confirmed by right renal angiography (Fig. 3A). No subsequent progression of anemia or the deterioration of renal function was observed after successful coil embolization (Fig. 3B): eGFR of 7.4 and 10.4 mL/min/1.73m^2^ 2 days and 5 days after TAE, respectively.

PCD-CT; photon-counting detector CT.

VMI; virtual X-ray monochromatic image.

## Discussion

Minimizing contrast media in patients with renal dysfunction helps mitigate the risk of CI-AKI. Still, few papers indicate how much contrast media should be reduced for contrast-enhanced CT in patients with renal dysfunction. Nyman et al. reported a linear correlation between the Iodine-dose/GFR ratio and the frequency of CIN (contrast-medium-induced nephropathy) in the coronary investigation. In addition, they reported CIN-1 (defined as a rise in s-Cr within 48–72 h post-procedure of >44.2 mmol/l (0.5 mg/ dl) or >25%) frequency in the contrast-enhanced CT of 12.0% at an Iodine-dose/GFR ratio=1.1, i.e., similar to the probability of unspecified CIN in the coronary studies [2]. We determined the iodine dose by referring to this previous study. However, pseudoaneurysms may not be detected at this rate due to insufficient image contrast. Previous studies showed that low-tube voltage imaging and low-energy VMI on EID-CT may increase the attenuation of iodine while preserving image quality [[6], [7], [8]]. Higashigaito et al. [9] compared image quality between VMI generated by EID-CT and PCD-CT, and demonstrated that PCD-CT provided better contrast than EID-CT at 40-50 keV.

This case report is the first to successfully identify a renal pseudoaneurysm with only 7.4 gI of a contrast media, which was 82.6% lower than that in our standard protocol, using PCD-CT. Low-tube voltage imaging and low-energy VMI on EID-CT have been shown to reduce contrast media by 20-60 and 20%-70%, respectively [10,11]. In another study using PCD-CT, VMI demonstrated a 25% reduction in contrast media while preserving image quality [9]. Stephan et al. reported a case of abdominal aortic aneurysm in an 81-year-old female with CKD that could be delineated with contrast media of 9.47 gI by using PCD-CT and VMI [12]. These studies indicate the potential usefulness of PCD-CT and VMI for aortic abnormalities with renal dysfunction.

Our case suggests the clinical potential of VMI on PCD-CT with ultra-low-dose contrast media for diagnosing small vascular abnormalities.

## Patient consent

Informed consent was obtained for patient information to be published in this article.
